# Experimental Demonstration of Ship Target Detection in GNSS-Based Passive Radar Combining Target Motion Compensation and Track-before-Detect Strategies

**DOI:** 10.3390/s20030599

**Published:** 2020-01-21

**Authors:** Fabrizio Santi, Debora Pastina, Marta Bucciarelli

**Affiliations:** 1 Department of Information Engineering, Electronics and Telecommunications, Sapienza University of Rome, 00184 Rome, Italy; debora.pastina@uniroma1.it; 2 Sympas S.r.l., 00192 Rome, Italy; bucciarelli@sympas-srl.it

**Keywords:** passive radar, GNSS-based passive radar, maritime surveillance, long integration time, track-before-detect

## Abstract

This work discusses methods and experimental results on passive radar detection of moving ships using navigation satellites as transmitters of opportunity. The reported study highlights as the adoption of proper strategies combining target motion compensation and track-before-detect methods to achieve long time integration can be fruitfully exploited in GNSS-based passive radar for the detection of maritime targets. The proposed detection strategy reduces the sensitivity of long-time integration methods to the adopted motion models and can save the computational complexity, making it appealing for real-time implementations. Experimental results obtained in three different scenarios (port operations, navigation in open area, and river shipping) comprising maritime targets belonging to different classes show as this combined approach can be employed with success in several operative scenarios of practical interest for this technology.

## 1. Introduction

Passive radar sensors are a very promising alternative to conventional systems exploiting dedicated transmitters. As is well known, the lack of the transmitting section lowers the costs of the system and its maintenance and enables covert operations, noticeably without the need of frequency allocations. Moreover, as the passive radar sensors do not emit any electro-magnetic radiation, the system is totally ‘green’ and hence installable in places where heavy active radars are undesired for their harmful radiations. While numerous studies focused on the exploitation of terrestrial-based illuminators, such as FM radio, analog/digital television, cell phone, and Wi-Fi signals, see for example [1,2,3,4], much less attention has been paid to satellite illuminators. These have been traditionally regarded as not adequate for the passive radar use, due to their low power levels and/or because in some cases the satellite orbit can make the revisit time not suitable. However, satellite-based passive radars present some relevant benefits with respect to their terrestrial-based counterparts: Wider accessibility on the global scale, less sensitivity to multipath effects, signals not blocked by mountains, and not reliance on potentially vulnerable infrastructures. Over recent years, the update of the current satellite fleets and the plan of new missions stimulated a rising interest in the development of innovative system concepts and techniques for satellite-based passive radar applications [5,6,7,8,9,10,11,12,13,14,15,16,17,18,19].

One of the fields of application that can more enjoy the availability of signal sources in remote areas is the maritime surveillance. Maritime areas far from land, such as international waters and economic exclusive zones, cannot be reached by the radio waves emitted by ground-based transmitters, but are in the scope of few satellite sources. Suitable candidates are communication satellites such as Inmarsat and Iridium [13,14], broadcasting satellites in geostationary orbits such as Astra and Eutelsat (DVB-S) [15], and Global Navigation Satellite Systems (GNSS) [16,17,18,19], with each choice having its own merits and shortcomings in terms of radar operations. Navigation satellites represent the best choice in terms of coverage, with constellation designed to guarantee a persistent and global illumination, even at the poles. Furthermore, six to eight satellites simultaneously illuminate any point on Earth from different angles if a single GNSS constellation is considered; such numbers could rise up to 32 satellites with all the four global systems (GPS, GLONASS, Galileo, and BeiDou) in full capacity. Therefore, GNSS offers a unique combination among global coverage, complete reliability, and spatial diversity.

The works [16,17] are a first introduction to the concept of maritime moving target detection with a GNSS-based passive radar. The experimental results therein provided showed that detecting vessels at sea exploiting the reflections of navigation satellite signals is fundamentally possible, but the chances to reveal the target are low if the target is small and/or far from the receiver. In fact, the main shortcoming of this technology stays in the fact that GNSS are very low effective isotropic radiated power (EIRP) sources, making the observation of most targets low. To overcome this limitation, advanced signal processing techniques are needed.

From a conceptual point of view, the signal energy can be strengthened by extending the integration time. Open literature reports a number of approaches to realize long time coherent integration to improve the radar detection capability in spite of the migration of the moving target through the resolution cells [20]. Nevertheless, in the application under consideration, it has been experimentally demonstrated that the maximum coherent processing interval (CPI) cannot exceed 2–3 s due to the unpredictable fluctuation of the target reflectivity, not enough for detecting most of the ship targets of interest [16]. Therefore, hybrid coherent/non-coherent integration methods have to be considered. In particular, [17] presents a method able to realize integration over long dwells (tens of seconds) for GNSS-based maritime surveillance. The idea behind this method is segmenting an overall dwell in short time intervals (defining the CPI and hereinafter named frames) and then performing a non-coherent combination of the signal energy pertaining the individual frames. (In this work, a ‘short’ time denotes an interval within which we can assume constant target reflectivity. As verified in a number of works [16,17,18,19], in the system under consideration this typically holds for intervals in the order of 2–3 s.) As the target is moving, the effective integration of the target energy over the whole dwell requires properly handling the migration through the resolution cells. To this purpose, a target motion compensation (TMC) procedure has been implemented. This assumes a linear variation of the target Doppler during the integration time and considers a bank of filters, each matched to a different admissible value of the Doppler rate, for compensating the range and Doppler migration. Such an approach has been shown able at improving the detection performance of the system, enabling the detection of targets undetectable with conventional short time approaches. Nevertheless, as TMC is a model-based procedure, it may suffer for motion model mismatches. This may be the case of a ship undergoing maneuvers, or the case in which the integration time has to be extended considerably.

Alternative strategies to handle energy integration over multiple frames belong to the class of track-before-detect (TBD) methods. The TBD paradigm consists in not declaring detections at each integration window, but rather feeding the tracker with unthresholded datastreams, operating integrations over possible trajectories, and finally declaring detections for those trajectories with high merit functions [21]. This type of approach opposes to the classical radar setup in which the tracker is subsequent to a detection (and possibly clustering) stage: By avoiding the hard decision taken at the detection level, information contained in the measurements is not discarded, increasing the capability to detect weak target echoes. A number of TBD approaches for both active [22,23] and passive sensors [24,25] has been proposed over the years. However, TBD methods are generally quite expensive in terms of computational load and memory resource requirements, as the amount of data to be processed and stored at each epoch is large, which makes it hard to implement these techniques in real-time systems [26]. A practical solution to reduce the complexity and make it affordable for real-time operations is considering a pre-processing stage that reduces the amount of data in input to the TBD processor. One such a solution, [27], considers a two-stage architecture. The first stage is a detector and plot extractor (DPE) that provides for each epoch a list of candidate plots resulting from a thresholding procedure. The use of a low threshold at this stage allows achieving a richer set of candidate plots, potentially increasing the detection capability. The latter is the TBD processor, which correlates the plots over successive epochs thus forming a track. Each candidate plot is therefore confirmed only if it gives rise to a sufficiently reliable past history, i.e., depending not only on its strength at the current time but also on the strength of the past plots linked to it and the number of plots in the track. Noticeably, in this kind of architecture the TBD operates directly on plot-lists, thus not requiring any discretization of the target state space as most of TBD methods. This allows a negligible complexity increase with respect to the conventional radar processing making the approach suitable for its application in practical systems [28].

In this work, we address the ship detection problem in the GNSS-based passive radar by combining the TMC with this type of two-stage TBD strategy. The idea is operating a TMC procedure over a limited number of frames, such that the motion model has higher chances to hold. Although the integration gain achieved over few frames could not suffice for strengthening the returns enough, setting a low decision threshold allows increasing the probability of their detection. Obviously, the false alarm rate will increase consequently, making the detections not reliable. The subsequent TBD processing will exploit the space-time correlation among the plots to discard the not reliable detections. In other words, the TBD processor acts as a filter to separate the false from the true target detections. The main goal of this paper is to give experimental evidences of the possibility of fruitfully exploiting such a combined detection strategy for the detection of maritime targets with the GNSS-based passive system (some preliminary results along this line were previously reported in [29]). To this purpose, we make use of real datasets collected in three different experimental acquisition campaigns carried out in different operative scenarios and using Galileo transmitters of opportunity. Ship targets belonging to different dimensional classes and undergoing different types of motion have been considered, thus verifying the wide applicability of the method in a number of operative conditions of practical interest.

This paper is organized as follows. Section 2 provides an overview on the GNSS-based passive radar for maritime surveillance, while the adopted combined detection strategy is detailed in Section 3. Experimental results are presented and discussed in Section 4. Section 5 closes the paper.

## 2. GNSS-Based Passive Radar for Maritime Surveillance

**Figure 1:**
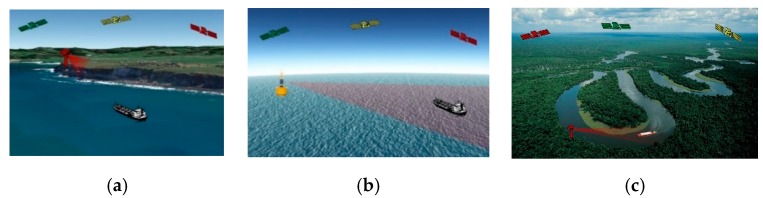
System concept examples of applications: (**a**) littoral traffic monitoring; (**b**) open sea surveillance; and (**c**) river traffic control of remote areas.

The operative conditions are given by navigation satellites as illuminators of opportunity and a passive receiver to observe remotely the sea area of interest. The receiving hardware could be mounted on the land, for littoral traffic monitoring or for supporting port operations; it could be also mounted on a moored buoy for offshore surveillance such as sea boarder monitoring or marine trade route controls. Figure 1 shows some examples of application of this technology.

**Figure 2:**
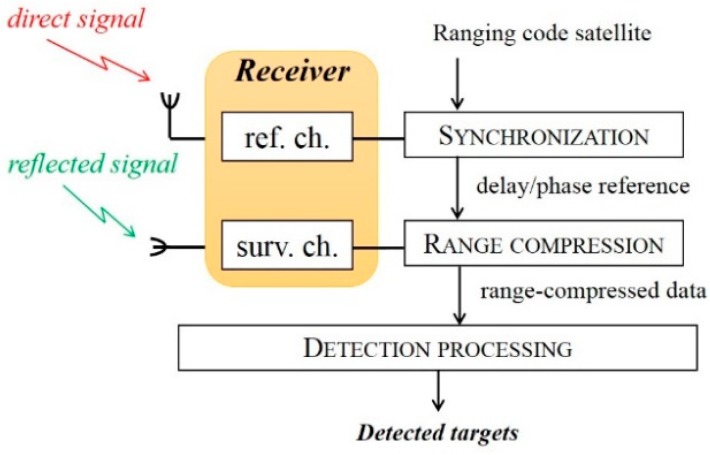
Processing scheme.

Figure 2 shows the high-level block diagram of the signal processing. As it can be seen, the receiver is equipped with two RF channels. One uses a low-gain antenna pointed toward the sky to record the direct signals (reference channel), while the latter operates with a high-gain antenna pointed toward the surveyed area to collect the reflections (surveillance channel). In most of passive radars based on terrestrial illuminators, the reference and surveillance signals are cross-correlated to perform range-compression. However, in the GNSS case the very low-power level at the input of the receiving antenna makes the reference channel too noisy, preventing its direct exploitation for matched filtering. Therefore, a signal synchronization stage is needed. This essentially tracks the direct signal parameters: Time delay, phase, Doppler frequency, and navigation message (if one exists). Then, by exploiting the full knowledge of the transmitted pseudo random noise (PRN) code, a noise-free replica of the direct signal is built, enabling a high-performance range compression [16].

As the GNSS signals are continuous-wave, a reformatting according to an equivalent pulse repetition interval (PRI) is previously performed. Usually, this is selected equal to 1 ms, i.e., the typical length of the PRN code. Let u be the variable defining the slow-time and $T_{obs}$ the observation time, so that $u\in \left[-\frac{T_{obs}}{2},\frac{T_{obs}}{2}\right]$ with step PRI. Let us assume a ship navigating in the radar field of view (FOV) during $T_{obs}$. Denoting with $\tau_{0}\left(u\right)$ and $\tau_{r}\left(u\right)$ the instantaneous delay times pertaining the direct and reflected paths, respectively, the differential bistatic range pertaining the target position at instant u is given by:

(1) $$r\left(u\right)=\frac{1}{c}\left[\tau_{r}\left(u\right)-\tau_{0}\left(u\right)\right],$$

c being the speed of light. Consequently, the instantaneous Doppler is given by:

(2) $$f_{d}\left(u\right)=-\frac{1}{\lambda }\left[\frac{1}{c}\frac{\partial \tau_{r}\left(u\right)}{\partial u}-\frac{1}{c}\frac{\partial \tau_{0}\left(u\right)}{\partial u}\right],$$

where λ denotes the wavelength.

In passive radar, the goal of the detection processing is identifying the moving target (or multiple targets) and extracting its (their) bistatic range and Doppler position(s). The classical approach to detect the target is applying a Doppler filtering over a short time interval of the range-compressed data. In practice, this corresponds to apply a Fast Fourier Transform (FFT) over an individual frame of duration $T_{f}$ (also defining the CPI) and detect peaks in the relative range and Doppler (RD) plane, usually by means of 2D Cell Average-Constant False Alarm Ratio (CA-CFAR) schemes. However, in the system under consideration the low-power level can make ineffective such an approach. To give an insight about this point, the following analysis has been conducted.

**Figure 3:**
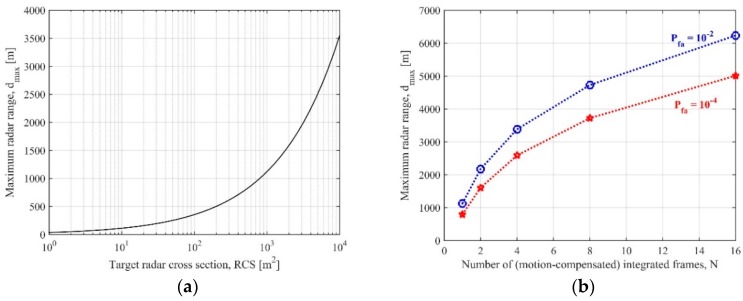
Maximum radar range for $P_{d}\geq 0.9$ versus (**a**) target radar cross section (RCS) in the single frame case with $P_{fa}\;=\;10^{-4}$; (**b**) number of integrated frames in the multi frame case with $P_{fa}\;=\;10^{-4}$ and $P_{fa}\;=\;10^{-2}$ (RCS = 27 dBm^2^).

Let us consider a receiving system operating with a surveillance antenna with gain $G_{rx}$, noise figure F, noise temperature $T_{0}$, and system losses $L_{s}$. As illuminator of opportunity, we refer to the Galileo E5a transmission, providing a power density on the ground $P_{DenGr}$ equal to $3\times 10^{-14}$ W/m^2^ and with carrier frequency 1176.450 MHz [30]. For a target characterized by radar cross section (RCS) $\sigma_{t}$, the maximum distance from the receiver at which it can be detected is given by:

(3) $$d_{max}=\sqrt{\frac{P_{DenGr}\;\sigma_{t}\;G_{rx}\;\lambda^{2}\;T_{f}}{{\left(4\pi \right)}^{2}\;SNR_{obj}\;k\;T_{0}\;F\;L_{S}}},$$

where $SNR_{obj}$ is the needed signal-to-noise ratio (SNR) in the RD map to assure a given probability of detection for false alarms not exceeding a desired rate. Figure 3a shows the maximum radar range for different values of the target RCS and system parameters reported in Table 1 (which are well in line with the experimental campaigns described in Section 4). $T_{f}$ has been set equal to 2.5 s and $SNR_{obj}$ has been evaluated by modeling the target as a Swerling I embedded in a white Gaussian background, maximum probability of false alarm $P_{fa}$ equal to 10^−4^, and probability of detection $P_{d}$ greater than 90% (corresponding equations can be found in [19]). As it is apparent, $d_{max}$ is strongly limited for targets belonging to the smallest dimensional classes. As example, to get reliable detections at ranges greater than 1 km we need to observe a target with RCS greater than 30 dBm^2^. To extend the detection range, longer integration times can be considered. Nevertheless, as aforementioned, extending the CPI is not practical due to the unpredictable fluctuations of the complex reflectivity. To overcome this limitation, a non-coherent combination can be considered, by performing a quadratic integration of the RD maps pertaining N successive frames.

Figure 3b shows as the detection range increases by considering the multi-frame integration (target RCS = 27 dBm^2^ has been selected as example). For example, for $P_{fa}\leq 10^{-4}$ and $P_{d}\geq 90\%$, such a target could be detected up to 5 km by integrating N = 16 frames (i.e., overall dwell equal to 40 s), thus increasing about five times the detection range with respect to the conventional single frame approach. However, as the target and the satellite are both moving, to accumulate effectively the target energy over the different frames a proper compensation of the range and Doppler migration has to be performed prior the quadratic integration. The curves in Figure 3 implicitly assume a perfect alignment of the target energy over the overall dwell (namely, motion-compensated frames have been assumed). However, this can be hard to be realized in the case of maneuvering targets and/or very long integrations. In fact, the compensation of the range and Doppler migration in spite of the unknown target motion is usually obtained by resorting to proper banks, based on proper assumed model(s) for the target motion(s) and being each branch in the bank matched to a specific motion condition represented by specific values of the model parameters. Such a strategy may require a large number of filters in the bank to take into account all the possible sets of motion.

To reduce the number of filters, the common practice is selecting a subset of values by making simplified assumptions on the variety of the observable motions. This can cause significant losses if the actual target motion sensibly deviates from the model, which can likely occur in the case of long integration times (namely, large N values).

An alternative solution to increase the detection range while keeping the number of frames limited, is combining the RD maps pertaining multiple satellites before the thresholding to reinforce the total target power, as proposed in [19]. However, this requires a demanding procedure in order to align the target energy as viewed in the different bistatic planes.

A different solution is considering a higher $P_{fa}$, which needs a lower detection threshold thus lowering the $SNR_{obj}$ in (3). For example, in Figure 3b we can observe that 16 frames are needed to detect the target at 5 km for $P_{fa}\;=10^{-4}$, while approximately the same distance can be covered by using half the number of frames if the false alarm rate increases by two orders of magnitude. As is obvious, such a strategy makes unreliable the decisions for the large number of false detections. However, the false measurements can be discarded by exploiting the space-time correlation among the detections occurred at different times. In the following section, we describe a detection strategy combining a model-based motion compensation method with a TBD approach to improve the detection capability of the system making it robust to motion model mismatches.

## 3. Detection Strategy

**Figure 4:**
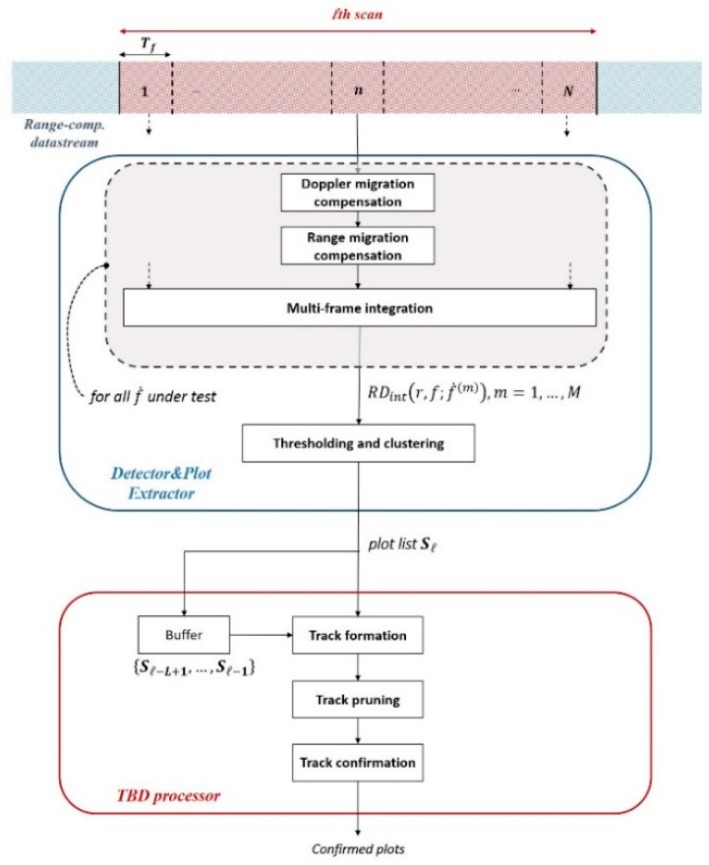
Detection scheme.

The adopted detection scheme is composed by two main stages: A DPE stage followed by the TBD processor [27]. At each epoch, the DPE receives in input the stream of range-compressed and slow time data and provides in output a list of plots by means of a TMC-based procedure. The plots pertaining the different epochs, hereinafter referred as to scans, are subsequently correlated by the TBD processor. Particularly, a list of plots is provided for each scan and the TBD correlates plots over L scans. As it will be clearer later, a scan is defined as composed by N≥1 frames of fixed duration $T_{f}$. Figure 4 depicts the block diagram of the detection processing, which is detailed in the following.

### 3.1. Target Motion Compensation Technique

As aforementioned, to extend the integration time in spite of the unpredictable target reflectivity fluctuations, a hybrid coherent/non-coherent procedure has to be implemented. This considers a multi-frame integration after a proper compensation of the target motion, which can be obtained by resorting to bank of filters according to suitable sets of possible kinematics parameters. To this purpose, several multi-frame integration methods consider the target range history as having a quadratic behavior [11,17], i.e.,

(4) $$r\left(u\right)\approx r_{0}-\lambda fu-\lambda \dot{f}\frac{u^{2}}{2},$$

(5) $$f_{d}\left(u\right)\approx f+\dot{f}u$$

being $r_{0}$, f, and $\dot{f}$ the bistatic range, Doppler centroid, and Doppler frequency rate at the reference instant u=0, respectively.

The basic-plane technique described in [17] implements a TMC by means of the following procedure. N successive frames of duration $T_{f}$ are selected from the range-compressed and slow-time datastream. Let n be the frame index, the nth frame undergoes the following steps:

- Doppler migration compensation—the assumed motion model entails a linear variation of the target Doppler (5), therefore the frame to frame Doppler migration is given by $\Delta f=\dot{f}nT_{f}$. Moreover, Doppler migration can occur even inside the individual frame and it can be described by the law $\delta f=\dot{f}u^{\prime }$, $u^{\prime }$ being the slow time variable spanning the individual frame. Therefore, both the Doppler migration inside each frame and frame to frame are both related to $\dot{f}$. These can be compensated by applying a slow time phase ramp comprising both sources of migration.
- Range migration compensation—this step is fed by the data in the fast-frequency and Doppler domain. Therefore, after the Doppler migration compensation, a slow time FFT and a fast time FFT are implemented. While range migration inside each frame can be neglected due to the coarse range resolution (not higher than 15 m for the GNSS signals with largest bandwidth, e.g., Galileo E5a/b or GPS L5), the frame to frame migration must be compensated. Form (4), the frame-to-frame range migration is composed by a range walk and a range curvature component, respectively described as $\Delta r_{walk}=-\lambda fnT_{f}$ and $\Delta r_{curv}=-\lambda \dot{f}\frac{{\left(nT_{f}\right)}^{2}}{2}$. The term $\Delta r_{walk}$ depends on the Doppler position and therefore it can be adaptively compensated by applying a fast frequency phase ramp for each Doppler bin, while $\Delta r_{curv}$ can be compensated by using a phase ramp according to $\dot{f}$.
- Motion-compensated RD map formation—a fast time inverse FFT allows finally achieving the motion compensated RD map.

It should be noticed that the procedure described above is completely adaptive except for the target Doppler rate. Therefore, a bank of M filters according to a grid of possible Doppler rates has to be considered. For the mth branch of the bank and for each frame, a motion compensated $RD_{n}\left(r,f;{\dot{f}}^{\left(m\right)}\right)$ map is obtained. The maps pertaining the different frames and the same value of the Doppler rate are then integrated in the intensity domain, so that at the output of the procedure a stack of integrated maps $RD_{int}\left(r,f;{\dot{f}}^{\left(m\right)}\right)$ with m=1,…,M is provided. In the ideal case of a target undergoing a motion perfectly matching the model in (4) and (5), the highest integration gain is obtained in the branch of the bank pertaining the actual Doppler rate, where the target has the highest chance to be detected.

The design of the bank obviously requires a proper setting of the Doppler rates to be tested. In order to guarantee that the uncompensated Doppler variation among the integrated frames is completely contained in the Doppler cell, the sampling of the Doppler rate axis must respect the following rule:

(6) $$\delta \dot{f}\leq \frac{1}{N\;T_{f}^{2}},$$

while the bounds can be set according to the maximum possible velocity.

Each map of the stack is finally subject to a thresholding procedure according to a desired level of $P_{fa}$. A clustering procedure is then applied. As depending on the input signal-to-noise ratio, the same target could be detected on more branches (i.e., the actual branch and some adjacent ones yielding ambiguities), the clustering stage also implements a post detection logic for ambiguities removal.

This approach has been proved able at increasing the ship detection capability of the GNSS-based passive radar system [17]. Nevertheless, the following issues should still be pointed out:

(i) As the procedure is based on a modelling of the motion, the performance can degrade in the case of model mismatches. Moreover, the model implicitly assumes the target as point-like. It should be noted that the sizes of ship targets of interest could be greater than the range resolution cell and that, depending on the particular motion conditions, the target energy could spread over multiple Doppler cells [31]. Because of the temporal variation of the target electro-magnetic response, it cannot be guaranteed that the strongest scattering point is confined to the same resolution cell in the motion-compensated maps pertaining the different frames. As the multi-frame integration is performed on a pixel basis, the highest integration gain could not be reached.
(ii) For given bounds of the Doppler rate to test, the sampling rule (6) implies that the number of filters in the bank increases with the number of integrated frames, entailing a direct increase of the computational load.

A straightforward solution to overcome these issues is limiting the integration at few frames, so that the motion model has higher chances to hold and the number of filters in the bank is such that the computational load is still affordable. As low N values could not suffice to sufficiently strengthen the target energy, to ameliorate this, the threshold can be set lower than usual, causing an increment in both $P_{d}$ and $P_{fa}$. The false measurements can be then rejected by the TBD processor described in the following sub-section.

### 3.2. Track-before-Detect Processor

Let us consider the TMC and multi-frame integration performed over N frames. These compose a scan. After detection and clustering, the processing of the ℓth scan provides in output a plot list:

(7) $$S_{\ell }=\left\{s_{1,\ell },\ldots ,s_{D_{\ell },\ell }\right\},$$

where $s_{k,\ell }$ is the vector containing the measured range and Doppler position, the Doppler rate pertaining to the map of the stack where the detection occurred, and the observed intensity:

(8) $$s_{k,\ell }=\left[r_{k,\ell },f_{k,\ell },{\dot{f}}_{k,\ell },I_{k,\ell }\right],$$

being $k=1,\ldots ,D_{\ell }$, namely the ℓth scan gives rise to $D_{\ell }$ detections.

**Figure 5:**
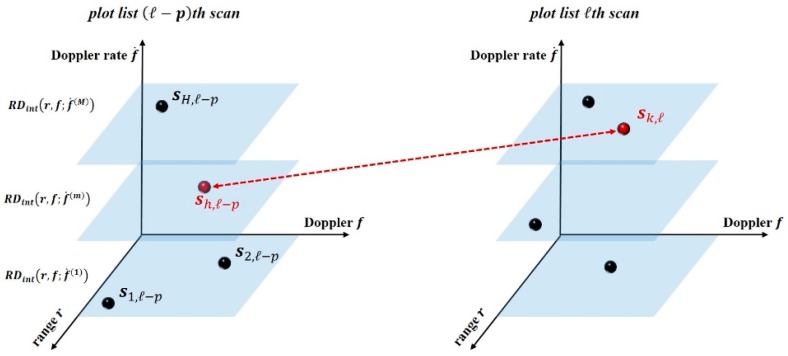
Correlations of plots belonging to successive plot lists.

By performing the procedure over successive scans, the TBD processor receives in input the current plot-list and correlates it with the past L−1 lists to form prospective tracks, as depicted in Figure 5. In the following, ΔT denotes the temporal shift between two successive scans.

The basic structure of the TBD processor implements the dynamic-programming algorithms defined in [27], but here properly modified and specialized for the considered framework. Additional developments/improvements could also be considered by incorporating strategies to deal with closely spaced targets and to further reduce the computational load [32,33]. The processing comprises the three main stages described below.

#### 3.2.1. Track Formation

The track formation stage correlates the plots taken from L successive plot lists to form a track, which can be defined by a vector $v=\left[v_{1},\ldots v_{L}\right]$. The ℓth entry of v is the index of the kth plot in the list $S_{\ell }$ belonging to the track. To take into account the possibility that the target is not detected in some scan, $v_{\ell }$ can be equal k=0 (missing observation) or $k\in \left\{1,\ldots ,D_{\ell }\right\}$ ($s_{k,\ell }$ belongs to v). To be admissible, v must not contain more than P consecutive null entries.

The formation of admissible tracks requires the definition of physical constraints on its evolution. Let $s_{h,\ell -p}$ be the last not null entry of an initialized track, with 1≤p≤P+1, its expected range and Doppler positions at the ℓth scan are given by:

(9) $${\hat{r}}_{h,\ell }=r_{h,\ell -p}-\lambda \;f_{h,\ell -p}\;p\Delta T-\lambda \;{\dot{f}}_{h,\ell -p}\frac{{\left(p\Delta T\right)}^{2}}{2},$$

(10) $${\hat{f}}_{h,\ell }=f_{h,\ell -p}+{\dot{f}}_{h,\ell -p}\;p\Delta T.$$

As no measurement of the Doppler rate variation is provided in the plot list, the expected Doppler rate can be defined as a value belonging to an interval defined according to the maximum expected target velocity and acceleration. Denoting these as $V_{Max}$ and $A_{Max}$, respectively, this constraint can be expressed as:

(11) $$\left|{\dot{f}}_{k,\ell }-{\dot{f}}_{h,\ell -p}\right|\leq \frac{3\;V_{Max}\;A_{Max}\;}{\lambda \;r_{h,\ell -p}}p\Delta T.$$

Therefore, the current plot $s_{k,\ell }$ can be linked to the plot $s_{h,\ell -p}$ if (11) and

(12) $$\frac{r_{k,\ell }}{{\hat{r}}_{h,\ell }}\in \left[1-m_{r},1+m_{r}\right],$$

(13) $$\frac{f_{k,\ell }}{{\hat{f}}_{h,\ell }}\in \left[1-m_{d},1+m_{d}\right]$$

hold, $m_{r}$ and $m_{d}$ being a number of range and Doppler cells, respectively, accounting for proper margins. In the case of multiple compatible plots, $s_{k,\ell }$ is linked with the one providing the highest intensity.

Each track is also scored by a vector storing the intensities of the individual plots. In the case of a missed detection, such a vector is filled with a null entry in the corresponding position.

#### 3.2.2. Track Pruning

After the track formation, it could happen that some plots belong to multiple tracks. To solve this ambiguity, a pruning of the track is implemented, which assigns the common plots only to the track exhibiting the highest score defined as the sum of the intensities of the individual plots belonging to the track.

#### 3.2.3. Track Confirmation

The pruned tracks formed by a number of plots lower than Q are considered not reliable and therefore deleted. Moreover, the survived tracks with a too low overall intensity may be deleted according to a further thresholding. After this procedure, the false measurements will be likely discarded.

Some final considerations are now in order:

(1) It could be noticed that the kinematic rules for plots correlation are still based on a linear Doppler model, as in the TMC. However, this model has to hold only inside each scan, while the technique allows to integrate returns from plots having different Doppler rates (because of (11) and as depicted in Figure 5). This allows a higher degree of freedom in performing energy integration among the overall observation interval.
(2) The integration is no more constrained by the resolution cells, as the TBD processor links plots having the highest intensity within an admissible RD area.
(3) The inclusion of proper margins in range and Doppler further relaxes the impositions on the target kinematics, so that the processor can handle deviations between the theoretical and actual motion model, including the presence of multiple scattering centers in the case of extended targets.

## 4. Experimental Verification

In this section, it is shown as the proposed strategy can be fruitfully exploited in scenarios of practical interest. To this purpose, real data collected during three experimental campaigns have been used. The receiving hardware used during the acquisition was a superheterodyne receiver mounted onto a van, which was also equipped with an automatic identification system (AIS) receiver to register in real time the actual locations of ship targets of opportunity. (The photographs and the information about the size of the ships of opportunity reported in this section have been taken from [34].) During all the experiments, the signals transmitted by Galileo satellites in the E5a band (carrier frequency: 1176.45 MHz), transmitting PRN codes with chip-rates equal to 10.23 MHz, have been acquired. During the synchronization, both the pilot and the data channels E5a-Q and E5a-I have been used.

### 4.1. Scenario 1: Port Operations

**Figure 6:**
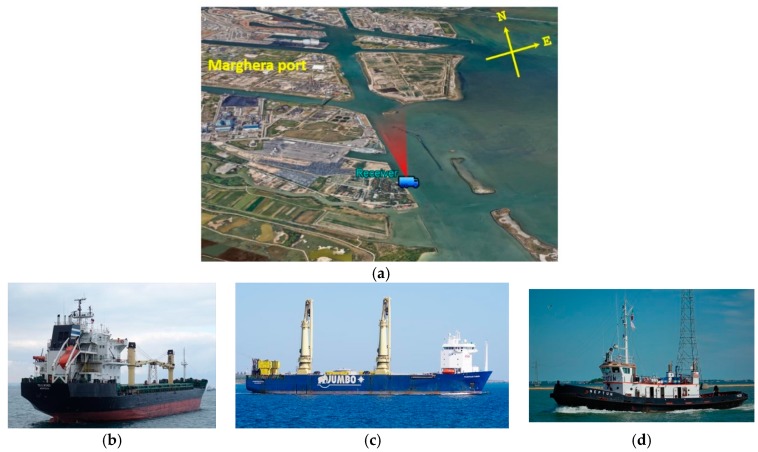
Marghera Port: (**a**) acquisition geometry; (**b**) Tailwind (cargo); (**c**) Fairpartner (heavy load carrier); and (**d**) Neptun (tug).

A first acquisition campaign was carried out near the Marghera port, Italy. The receiver was placed at the entrance of the port area, with the radar antenna pointed toward the terminal area, as depicted in Figure 6a. During the acquisition of about 3 min, the three targets shown in Figure 6b–d were in the FOV. The cargo Tailwind, with size (length overall by breadth extreme) 149.4 m by 23 m and the heavy load carrier Fairpartner (size: 143.1 m by 26.6 m) were entering in the port area. At the beginning of the acquisition, Tailwind was already in the FOV, remaining in it for the whole acquisition, while Fairpartner entered in the FOV after about one and a half minute. Moreover, at the end of the acquisition the tug Neptun (29.37 m by 8.5 m) was leaving the terminal area entering in the FOV and approaching the receiver.

The experimental and signal processing parameters are listed in Table 2. Particularly, the DPE stage operates TMC and multi-frame integration over intervals of 4 × 2.5 s = 10 s. Detections are declared according to thresholds to guarantee $P_{fa}$ equal to 10^−4^, 10^−3^, or 10^−2^. The corresponding plot lists are sent to the TBD processor that uses L = 8 scans to confirm the detections. As the interval between two scans is set equal to 5 s, the overall dwell to get the final decision is 45 s. It should be also pointed out that, in this particular scenario, the targets move along an a priori known direction, as all the ships follow the route to entry/exit the port area. As the receiving antenna boresight is aligned onto this direction, it is expected that all the targets exhibit a dominant radial motion, namely a negligible Doppler rate. Therefore, in these particular conditions, the TMC has been performed by considering a priori known and equal to zero the value of the Doppler rate.

**Figure 7:**
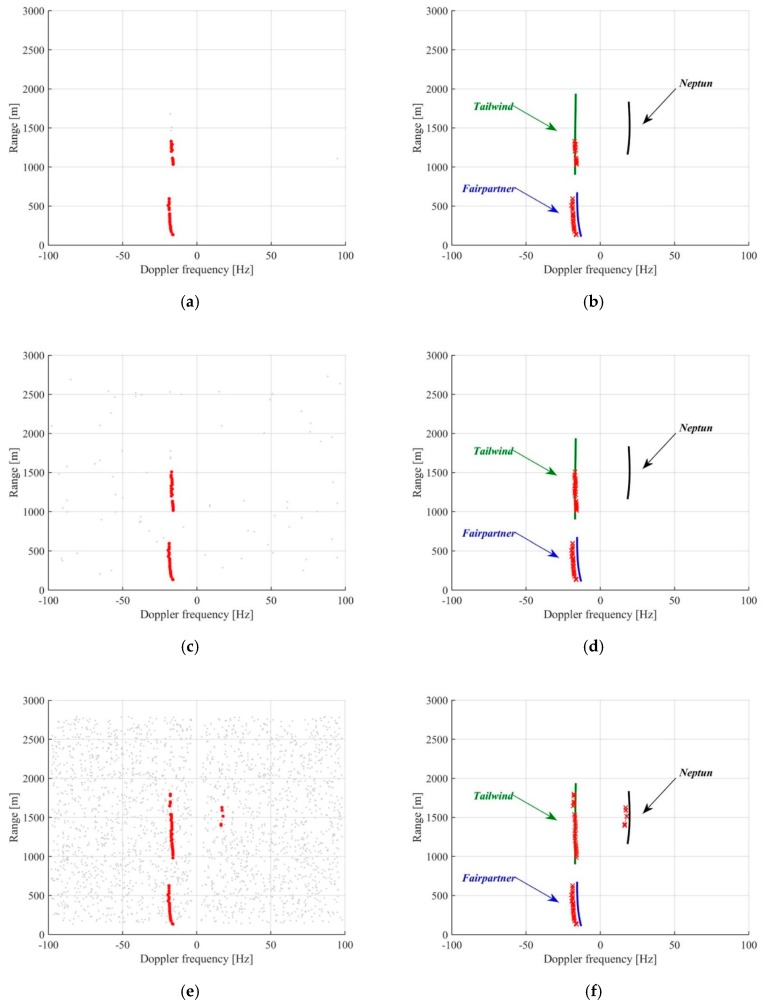
Marghera port detection results - plot lists over the different scans for detector and plot extractor (DPE) operating with $P_{fa}\;=\;10^{-4}$ (**a**), $10^{-3}$ (**c**), and $10^{-2}$ (**e**); comparison between the resulting tracklets and the automatic identification system (AIS) ground truth (full lines) for $P_{fa}=10^{-4}$ (**b**), $10^{-3}$ (**d**) and $10^{-2}$ (**f**).

Figure 7 shows the detection results. Particularly, Figure 7a,c,e are the superimpositions of the plot lists obtained over the successive integration windows for $P_{fa}$ levels equal to 10^−4^, 10^−3^, and 10^−2^, respectively. The greater number of alarms achieved lowering the threshold is easily observed. In these figures, the red markers denote the plots confirmed after the TBD processing: It can be observed as the false measurements have been correctly discarded, while the surviving plots form tracklets that are compared with the AIS ground truths in Figure 7b,d,f. The following comments apply:

- Fairpartner has been correctly detected in almost all the epochs in which it was in the FOV for all the considered $P_{fa}$. This is not an unexpected result, as this is a massive target very close to the receiver so that it has a high detection probability even for low $P_{fa}$. However, even if small, an increase of the detection rate could be shown lowering the threshold: In the 22 timestamps in which it was in the FOV, it has been detected 20, 21 and 22 times for $P_{fa}$ equal to 10^−4^, 10^−3^, and 10^−2^, respectively.
- The use of the lower threshold allowed detecting Tailwind (which is at a higher distance than Fairpartner) for a longer interval, as it can be easily observed by comparing the tracklets in Figure 7b,d,f. Particularly, it was detected at longer ranges with $P_{fa}$ = 10^−2^ and evaluating the detection rate as the number of scans in which it gave rise to a confirmed detection over the overall scans, we move from 33% and 58% for $P_{fa}$ = 10^−4^, 10^−3^, respectively, up to 89% when $P_{fa}$ = 10^−2^.
- Neptun is a small target, which has small chances to be detected. In fact, with conventional processing involving $P_{fa}$ values equal to 10^−3^ and 10^−4^, it has not been detected. Nevertheless, using the proposed approach with a lower detection threshold ($P_{fa}$ equal to 10^−2^), it was nicely detected.

### 4.2. Scenario 2: Navigation in Open Area

**Figure 8:**
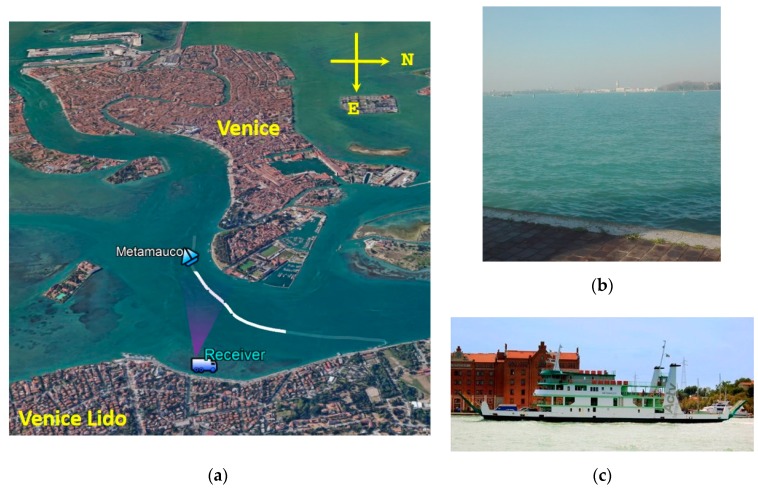
Venice lagoon: (**a**) acquisition geometry; (**b**) optical photograph of the surveyed area; and (**c**) Metamauco (ro-ro ferry).

A second acquisition campaign was carried out at the Venice lagoon, Italy. The receiver was placed on the West side of the Venice Lido Island with the surveillance antenna pointed toward the waterway crossing the lagoon [see Figure 8a,b]. During an acquisition of several minutes, the ro-ro passenger car ferry Metamauco shown in Figure 8c (size: 57.85 m by 13.1 m) entered in the FOV. As it can be inferred from the AIS ground truth [white line in Figure 8a], for this target, a constant radial velocity cannot be expected. Actually, it should be point out that this scenario aims at simulating the situation in which the receiver is placed in an open area, where specific routes cannot be predicted.

The experimental and signal processing parameters for this acquisition are listed in Table 3. In this case, two operating modes have been considered for the DPE stage. In the former, the TMC considers only the migration compensation accounting the null value of the Doppler rate, which corresponds to assume a constant Doppler over the integrated frames. In the latter, the TMC is performed according to the whole set of admissible Doppler rates. Moreover, $P_{fa}$ level at the DPE stage is set equal to 10^−4^ and 10^−2^.

**Figure 9:**
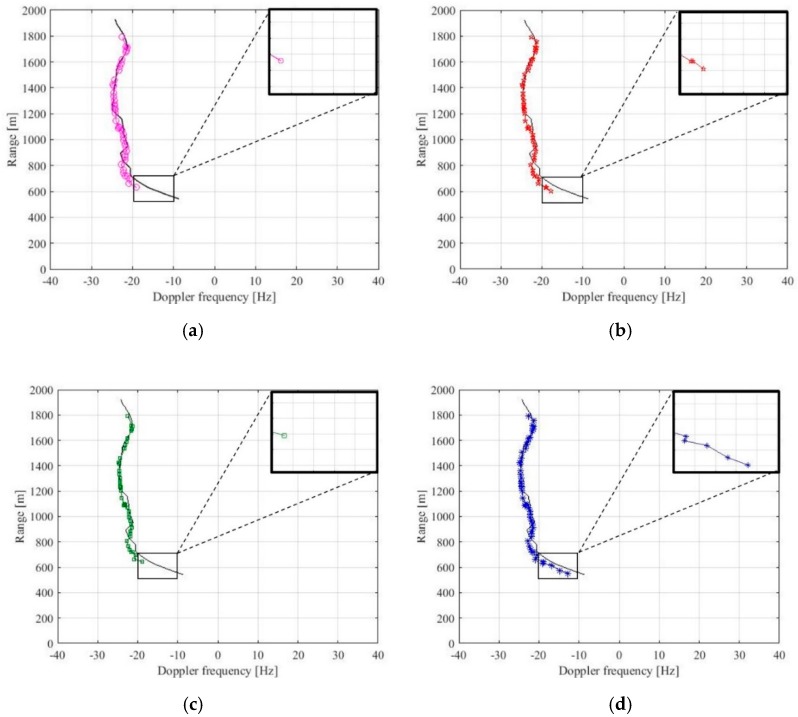
Venice lagoon detection results after track-before-detect (TBD) for different DPE operating modes: (**a**) target motion compensation (TMC) only for $\dot{f}=0\;\mathrm{Hz}/s$, $P_{fa}=10^{-4}$; (**b**) TMC only for $\dot{f}=0\;\mathrm{Hz}/s$, $P_{fa}=10^{-2};$ (**c**) TMC with full bank, $P_{fa}=10^{-4}$; and (**d**) TMC with full bank, $P_{fa}=10^{-2}$.

Figure 9 shows the detection results after the TBD processor. The full lines represent the Metamauco AIS track, while the markers are the final detections. As it can be seen, in both the TMC operating modes and for both the $P_{fa}$ levels considered, the target is correctly detected for a long part of its trajectory where it shows a quite constant Doppler. The relevant part here where we focus our attention is the part of the track in the region highlighted by the black boxes. This part corresponds to the entrance of the target in the FOV, where it cannot benefit for the maximum antenna gain thus likely exhibiting a weaker echo. Moreover, the target undergoes a maneuvering motion, resulting in a varying Doppler. Comparing Figure 9 a,c we can see that the target is almost not detected for $P_{fa}=10^{-4}$, nor for the case of TMC with only null Doppler rate neither in the case of full bank. Lowering the threshold for $P_{fa}=10^{-2}$, a small improvement in detection capability can be appreciated in Figure 9b (TMC with $\dot{f}=0$ Hz/s only), while combining the use of the bank with the low thresholding [Figure 9d], the weak target return can be detected also during its maneuvers.

### 4.3. Scenario 3: River Shipping

**Figure 10:**
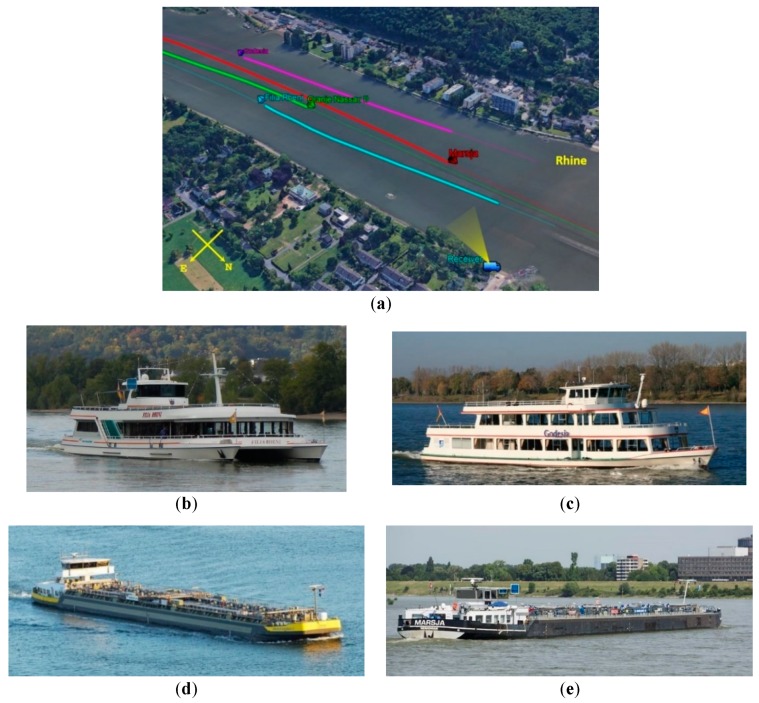
Rhine River: (**a**) acquisition geometry; (**b**) Filia Rheni (passenger ship); (**c**) Godesia (passenger ship); (**d**) Oranje Nassau II (inland motor tanker); and (**e**) Marsja (inland motor tanker).

A last experimental campaign has been carried out near Bonn, Germany. In this case, the receiver was placed on the shore of the Rhine observing the river traffic, mainly composed by barges and inland cruise ships. During a two mins acquisition, four targets were in the FOV. They were the passenger ships Godesia (38.6 m by 8.6 m) and Filia Rheni (42 m by 11 m), moving away from the receiver, and the two motor tankers Oranje Nassau II (110 m by 13.5 m) and Marsja (110 m by 11.44 m), approaching, whose AIS ground truths are shown as full lines in Figure 10.

Two Galileo satellites were simultaneously tracked so that the detection processing was performed for both the resulting bistatic geometries. For the TMC the full Doppler rate bank has been used and the $P_{fa}$ has been set equal to 10^−2^. The remainder of the processing parameters are listed in Table 4.

**Figure 11:**
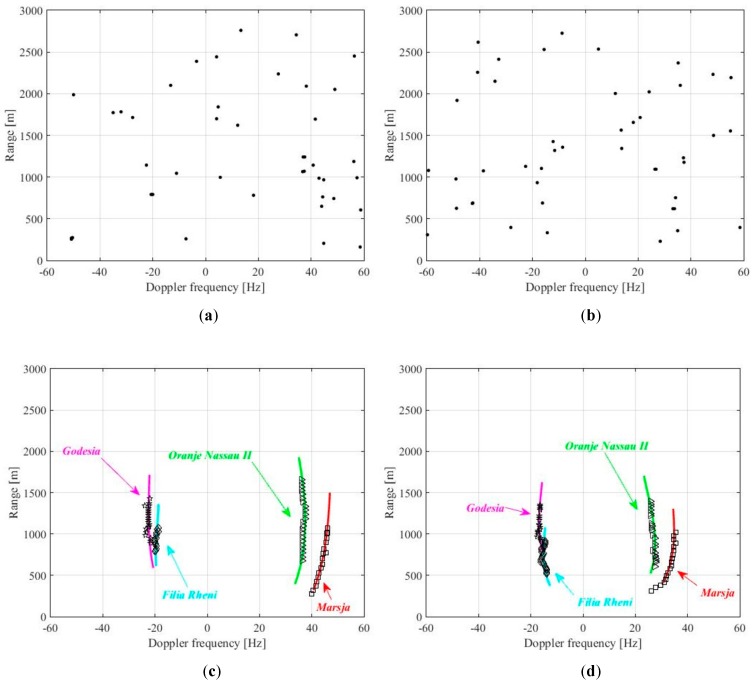
Rhine River detection results: (**a**) satellite 1 detection map for an individual scan; (**b**) Satellite 2 detection map for an individual scan; (**c**) satellite 1 TBD output; and (**d**) Satellite 2 TBD output.

Figure 11 shows the detection results for both the geometries. Particularly, Figure 11a,b show the set of detections obtained for the plot lists pertaining satellites 1 and 2, respectively, at a single scan time. Figure 11c,d show the TBD outputs for the whole observation interval. The nice agreement between the detected tracklets and the AIS ground truths (full lines) can be observed for both the small passenger ships and the barges in both the considered geometries.

It is worth to point out that in the case of multistatic acquisitions the system is potentially able to localize the targets. Usually, this is obtained via multilateration methods, consisting in intersecting bistatic range ellipsoids obtained at each epoch [18]. However, this can be demanding if the number of plots observed in the different geometries is large; moreover, the possibility to give rise to ghost targets located in the surveyed area, and therefore hardly to be discarded, grows consequently. The proposed method can be usefully exploited to alleviate such issues. In fact, the plot-to-plot correlation performed by the TBD processor at each bistatic level allows a subsequent Cartesian tracker to operate on a smaller number of highly reliable detections [35]. Alternatively, the localization task could be obtained by directly associating the bistatic tracklets in place of the individual plots, allowing to directly exploiting the evolution of the bistatic measurements to individuate the inadmissible combinations (i.e., the ghost targets). Finally, a challenging task that could be considered in the future is to make able the TBD processor to jointly process multistatic plot lists to return tracklets directly in the Cartesian plane.

## 5. Conclusions

This paper explored as using a two-stage detection strategy relying on target motion compensation and track-before-detect processor could be fruitfully exploited to enable maritime surveillance with a GNSS-based passive radar. The unfavorable power budget provided by navigation satellites requires in fact proper detection techniques to improve the detectability of ship targets of interest. The multi-frame integration performed over a limited number of motion compensated frames potentially allows target actual kinematic to fit to the motion model for the compensation of the range and Doppler migration. Although the integration gain achieved over such limited dwells could not suffice to strengthen the target energy sufficiently, the use of a low threshold can ameliorate this problem and allow the target detection. Exploiting the space-time correlation among successive plot lists, the subsequent TBD processor can filter out the false alarms, providing in output high reliable detections, furthermore with their temporal evolution.

The effectiveness of the approach has been tested in a number of experimental case studies, including different scenarios and targets belonging to different dimensional classes and undergoing different types of motion. The achieved results have shown as this detection strategy may increase the detection capability of the system under a variety of situations of practical interest.

Next stage of this research is exploring as the proposed framework can be used in the multistatic configuration (i.e., multiple satellite acquisitions) to ameliorate the localization performance of multilateration algorithms.

## Figures and Tables

**Table 1:** Receiving chain parameters.

Parameter	Symbol	Value
Power density on the ground	$P_{DenGr}$	3 × 10^−14^ W/m^2^
Antenna gain	$G_{rx}$	15 dB
Noise figure	F	1.5 dB
Losses	$L_{s}$	2 dB
Noise Temperature	$T_{0}$	290 K
Boltzmann constant	k	1.38 × 10^−23^ J/K

**Table 2:** Marghera port—experimental and signal processing parameters.

Parameter		Value
**Satellite**	Number	GSAT0208
	PRN code	E08
	Aspect angle (clockwise from North)	100°
	Elevation angle	57°
**Signal processing**	Frame duration T_f_	2.5 s
	Number of integrated frames N	4
	Number of scans for plot confirmation L	8
	Offset between consecutive scans ΔT	5 s
	Maximum consecutive missed detection P	3
	Minimum detections in a track Q	5

**Table 3:** Venice lagoon—experimental and signal processing parameters.

Parameter		Value
**Satellite**	Number	GSAT02014
	PRN code	E05
	Aspect angle (clockwise from North)	80°
	Elevation angle	55°
**Signal processing**	Frame duration T_f_	3 s
	Number of integrated frames N	3
	Number of scans for plot confirmation L	8
	Offset between consecutive scans ΔT	4.5 s
	Maximum consecutive missed detection P	3
	Minimum detections in a track Q	5

**Table 4:** Rhine River—experimental and signal processing parameters.

Parameter		Value
**Satellite 1**	Number	GSAT02010
	PRN code	E01
	Aspect angle (clockwise from North)	164°
	Elevation angle	9°
**Satellite 2**	Number	GSAT02014
	PRN code	E05
	Aspect angle (clockwise from North)	−116°
	Elevation angle	36°
**Signal processing**	Frame duration T_f_	2.5 s
	Number of integrated frames N	4
	Number of scans for plot confirmation L	8
	Offset between consecutive scans ΔT	5 s
	Maximum consecutive missed detection P	3
	Minimum detections in a track Q	5
